# Ru-Doped Induced Phase Engineering of MoS_2_ for Boosting Electrocatalytic Hydrogen Evolution

**DOI:** 10.3390/nano15100777

**Published:** 2025-05-21

**Authors:** Renjie Li, Meng Yu, Junjie Li, Ning Wang, Xiaolong Yang, Yanhua Peng

**Affiliations:** College of Chemistry and Chemical Engineering, Qingdao University, Qingdao 266071, China; lrj222629@163.com (R.L.);

**Keywords:** Ru-doped, MoS_2_, hydrogen evolution reaction, XAFS, alkaline conditions

## Abstract

Electrochemical hydrogen evolution reaction (HER) holds great potential as a sustainable strategy for green hydrogen production. However, it still faces significant challenges due to the lack of highly efficient electrocatalysts. Herein, a synergistic approach by incorporating Ru atoms into MoS_2_ nanosheets to optimize the structure and conductivity has been proposed, which could improve the HER performance of MoS_2_ under alkaline conditions. Combining theoretical calculations and structural characterizations, it is demonstrated that the Ru atom introduction leads to the localized distortions of MoS_2_, generating additional active sites for H* adsorption, and reduces the free energy to adsorb and desorb hydrogen. Furthermore, the Ru introduction makes partial transformation from the 2H phase to the 1T phase in MoS_2_, which results in the change of the electronic structure and further enhances the electrical conductivity. As a result, the Ru-doped MoS_2_ electrocatalysts exhibit the high HER activities with the low overpotentials of 61 mV and 79 mV at 10 mA cm^−2^ in 1.0 M KOH and alkaline seawater, respectively. This work provides a novel design strategy for enhancing HER activity through the synergistic modulation of structural and electronic properties, offering valuable insights for the development of efficient electrocatalysts for hydrogen evolution.

## 1. Introduction

The depletion of fossil fuels and the pollution of environment have prompted the world to look for alternative and green energy [1,2]. Hydrogen energy has been considered as the best option due to its zero-emission, high energy density, and environment-friendly nature [3,4]. Among numerous strategies for hydrogen production, the electrochemical HER has attracted widespread interest due to its high efficiency [5,6,7]. Obviously, the high-performance and cost-effective electrocatalysts are greatly essential for the efficiency of HER [8,9]. Usually, noble metal-based materials, especially Pt/C, are widely considered as efficient electrocatalysts [10,11]. However, their scarcity and high cost prevent them from widespread application in the industrial sector [12,13]. Therefore, it is desirable to create high-performance and cost-effective electrocatalysts for the efficient production of hydrogen.

Metal sulfide materials have been increasingly employed in hydrogen evolution reactions including electrocatalytic reactions and photocatalytic reactions [14,15]. Among transition metal sulfides, MoS_2_ has emerged as the most promising among potential electrocatalysts to replace platinum due to its exceptional ability to adsorb and desorb hydrogen at edge sites, high specific surface area, and superior chemical stability [16]. Generally, MoS_2_ predominantly exists in two phases in nature: the stable 2H-MoS_2_ and the metastable 1T-MoS_2_. Typically, the metallic 1T phase of MoS_2_ exhibits superior HER performance compared to the semiconductive 2H phase, which is attributed to its lower charge transfer resistance from catalysts to solutions [17,18,19]. To improve the electrocatalytic activity of 2H-MoS_2_, loading conductive materials such as graphene, carbon, and noble metals has been reported in previous studies [20,21,22,23]. Additionally, strategies such as chemical doping and the introduction of defects have also been employed to boost HER performance [24,25,26]. Moreover, phase junctions within the same materials have been demonstrated as a promising approach due to the unique benefits of robust coupling, high electron affinity, and the absence of foreign elements. For instance, Cai and coworkers demonstrated that the incorporation of 25% 1T-MoS_2_ into 2H-MoS_2_ nanosheets resulted in a ten-fold increase in electron concentration [27]. Most recently, we have reported a significant enhancement in photocatalytic hydrogen evolution through improving the conductivity of ZnIn_2_S_4_ by the incorporation of the 2H@1T-MoS_2_ phase [28]. However, the mechanism of the MoS_2_ transformation from 2H to 1T is still unclear, and the influence of the phase transformation of MoS_2_ on the HER activity also requires in-depth research.

Ruthenium (Ru) doping has been reported to promote the phase transformation of MoS_2_ from 2H to 1T and shows the high HER activity. Moreover, the bond energy of Ru-H is approximately 65 kcal mol^−1^, equivalent to the energy of Pt-H bond [29,30,31]. In previous studies, Ru materials including single atoms, nanoparticles, and clusters, have been demonstrated to possess high HER performances [32,33,34,35]. Zhang et al. have reported that MoS_2_-based electrocatalysts loaded with the single-atom Ru exhibit the significantly enhanced HER activity due to the 2H-to-1T phase transition [36]. The reason for phase transition is owing to the introduction of sulfur vacancies by Ru single-atom doping. Recently, Wang and co-workers believed that single-atom Ru substitution for the Mo atom in MoS_2_ resulted in the 2H-to-1T phase transition, which makes the Ru-MoS_2_ electrocatalysts have superb HER performances [37]. However, there is still controversy over the mechanism of the MoS_2_ transformation from 2H to 1T through the introduction of Ru. It is necessary to reveal the mechanism through the combination of theoretical calculation and experimental study.

In this work, we employed the DFT calculation and experiments to study the electrocatalytic HER activity on Ru-doped MoS_2_ catalysts. The theoretical calculations revealed that the introduction of Ru could cause the disordered structure of MoS_2_ next to Ru, which resulted in the 2H-to-1T phase transition. The experimental results supported the DFT calculation and suggest that Ru doping reduced the energy barrier to obviously decrease the overpotentials of HER. Consequently, the Ru-doped MoS_2_ show the overpotentials values of 61 mV and 79 mV at 10 mA cm^−2^ in 1.0 M KOH and alkaline seawater, respectively.

## 2. Materials and Methods

### 2.1. Materials

(NH_4_)_6_Mo_7_O_24_∙4H_2_O (AR.99%) and CS(NH_2_)_2_ (AR.99%) were purchased from Macklin Biochemical Co., Ltd., (Shanghai, China). KOH was acquired from Hengxing Chemical Company (Tianjin, China). RuCl_3_∙XH_2_O (35.0–42.0% Ru basis) was obtained from Aladdin BioChem Co., Ltd., (Shanghai, China). Absolute alcohol (AR) and sodium hydroxide were obtained from Sinopharm Chemical Reagent Co., Ltd., (Beijing, China). The supplier of carbon paper (CP) was Chuxi industrial Co., Ltd., (Shanghai, China). Nafion PFSA polymer (5%) was purchased from Suzhou Sinero Technology Co., Ltd., (Suzhou, China). All reagents employed were of analytical grade.

### 2.2. Apparatus

XRD measurements were conducted using a Smart Lab 3 kW diffractometer (Rigakum, Tokyo, Japan) with Cu Kα radiation. TEM imaging was conducted utilizing a Hitachi HT7700 instrument (JOEL, Tokyo, Japan). Raman spectroscopy was conducted at ambient temperature with a DXR2 Raman spectrometer (Thermo Fisher, Waltham, MA, USA), employing a 532 nm laser as the excitation source. XPS data were acquired using a Thermo ESCALAB Xi electron spectrometer (Thermo Fisher, Waltham, MA, USA), equipped with an Al Kα radiation source (hν = 1486.6 eV). The survey scans were conducted over an energy range of 0–5000 eV. The microstructure and morphology of samples were investigated using a scanning electron microscope (SEM, Regulus 8100) (JOEL, Tokyo, Japan). HAADF-STEM images were acquired on a JEOL JEM-ARM300 microscope (JOEL, Tokyo, Japan), operated at a beam energy of 200 keV. EDX, integrated within the HAADF-STEM, was utilized to obtain detailed elemental mappings. pH measurements were conducted using a pH meter from INESA (Shanghai, China). Sonication was carried out using a Supmile Ultrasonic Cleaner, sourced from Kunshan, China. Additionally, XAFS spectroscopy was conducted at the 1W1B beamline of Shanghai Synchrotron Radiation Facility (SSRF) in China.

### 2.3. Synthesis of Electrocatalysts

Synthesis of MoS_2_ electrocatalysts: 1.0 mmol of (NH_4_)_6_Mo_7_O_24_∙4H_2_O and 35.0 mmol of CS(NH_2_)_2_ were dissolved in 50.0 mL of deionized water. The solution was subjected to vigorous stirring at an ambient temperature to ensure the formation of a homogeneous mixture. The prepared solution was transferred into a Teflon-lined autoclave and subjected to a 24 h heating process at 180 °C. Following cooling to an ambient temperature, the black precipitate was collected through a washing and drying process.

Synthesis of Ru-doped MoS_2_ electrocatalysts: 40.0 mg of MoS_2_ power was dispersed in 25.0 mL of deionized water with continuous stirring. Subsequently, 0.04, 0.08, and 0.12 mmol of RuCl_3_∙XH_2_O was added to the MoS_2_ suspension during the stirring process. Following an 80 min sonication period, the homogenized solution was transferred into a Teflon-lined autoclave and then heated at 90 °C for 3 h. When the reaction was completed, the black precipitates were washed with deionized water and ethanol to remove any remaining solutes. Following vacuum drying at 55 °C, the product was obtained. The Ru-doped MoS_2_ nanosheets with varying concentrations of RuCl_3_ were designated as 0.04-Ru/1T@2H-MoS_2_, 0.08-Ru/1T@2H-MoS_2_, and 0.12-Ru/1T@2H-MoS_2_, respectively.

Synthesis of Pt/C/CP electrode: Prior to the experimental procedure, the carbon paper (CP) required preliminary treatment. Initially, the CP was subjected to an ultrasonic cleaning process for 30 min, sequentially in 0.10 M NaOH, 0.10 M HNO_3_, deionized water, and ethanol, respectively. Subsequently, the ultrasonically treated CP was allowed to air-dry overnight. A blend consisting of 5.0 mg of 20% Pt/C and 50 μL of 5% Nafion solution was dispersed in a mixture of 500 μL ethanol and 500 μL deionized water to form a uniform ink achieved by sonication. The prepared ink was coated onto a single piece (1 cm^2^) to fabricate a CP electrode coated with Pt/C.

Synthesis of working electrode: 5.0 mg of sample powder was mixed with 50 μL of a 5% Nafion solution, which was dispersed in a combined solvent of 500 μL ethanol and 500 μL deionized water. Then, the mixture was subjected to sonication for 1 h to achieve a dispersion. Finally, 20 μL of the dispersion was deposited onto the carbon paper, which was then dried at room temperature to fabricate the working electrode.

### 2.4. Electrochemical Measurements

These measurements were conducted using a three-electrode cell configuration, equipped with a CHI 660E electrochemical workstation (Shanghai Chenhua Instrument Co., Ltd., Shanghai, China). The SCE electrode and the Ag/AgCl electrode served as the reference electrodes, while a graphite flake was employed as the counter electrode. For all measurements, the SCE electrode was calibrated against the RHE standard. All potential values were referenced to the RHE using the calibration equations:

*E*_RHE_ = *E*_SCE_ + (0.241 + 0.059 × pH) (1)

*E*_RHE_ = *E*_Ag/AgCl_ + (0.210 + 0.059 × pH) (2)

Electrochemical measurements were carried out in 1.0 M KOH solution and alkaline seawater. All LSV curves were obtained at a scan rate of 5 mV/s. EIS was performed with a frequency spectrum from 0.01 Hz to 100 kHz using an alternating current (AC) amplitude of 5 mV. The measurements were carried out within a potential range of −0.8 V to −1.6 V (vs. SCE) in 1.0 M KOH solution, and from −0.8 V to −1.6 V (vs. Ag/AgCl) in alkaline seawater. Corresponding LSV data were analyzed by fitting them to the Tafel equation [38]:

*η* = a + b × log *j* (3)

where *η* is the overpotential, *j* is the current density, and b is the Tafel slope. The electrochemical double-layer capacitance (*C*_dl_) was determined and used to calculate the electrochemical active surface area (ECSA) through the application of cyclic voltammetry (CV) within a potential range that avoids faradaic reactions. We employed a series of scan rates (5, 10, 15, 20, 25 mV/s) for the cyclic voltammetry measurements. The capacitive current density was measured at the average potential. Subsequently, the C_dl_ was determined as the slope of the resulting linear fit. Typically, the specific capacitance falls within the range of 20–60 μF cm^−2^. We selected the average value of 40 μF cm^−2^ to provide a rough estimate of ECSA using the following equation [39]:

(4) $$\mathrm{ECSA}=\frac{Cdl}{{40\;\mu F\;\cdot cm}^{-2}}\;{\mathrm{cm}}^{2}$$

To assess the stability of the catalyst, a cycling stability test was conducted over a potential range from −0.8 V to −1.6 V (vs. Ag/AgCl) at a scan rate of 100 mV/s, spanning 1000 cycles. Additionally, a durability test was carried out at an overpotential of 10 mA cm^−2^ for a duration of 12 h.

### 2.5. DFT Calculation

First-principles calculations were conducted using the Materials Studio software (4.0), employing the PAW method [40]. The exchange-correlation functional was modeled using the PBE-GGA [41]. The (5 × 5) 2H-MoS_2_ (002) and (5 × 5) 1T-MoS_2_ (002) were depicted using periodic slab models, corresponding to the most thermodynamically stable facets [42]. A 15 Å-thick vacuum layer was introduced to avoid a spurious interaction between periodic boundary images and the convergence criteria for structural optimization were set at 0.02 eV/Å for force and 10^−5^ eV for energy precision.

∆*G*_H*_ for hydrogen adsorption on MoS_2_ surfaces was computed using the methodology introduced by Nørskov et al. [43].

(5) $$E_{ads}=E_{total}-(E_{surf}-E_{H_{2}}/2)$$

(6) $${∆G}_{H^{*}}=E_{total}-\left(E_{surf}-\frac{E_{H_{2}}}{2}\right)+{∆E}_{ZPE}-T∆S$$

where $E_{ads}$ is the adsorption energy of H, $E_{total}$ is the overall energy of the system when hydrogen is adsorbed, $E_{surf}$ is the energy of the bare, $E_{H_{2}}$ is the energy of hydrogen molecules, ${∆E}_{ZPE}$ is the alteration in zero-point energy, and ∆S is the entropy variation associated with the conversion from adsorbed H to H_2_ at standard atmospheric pressure.

## 3. Results and Discussion

### 3.1. Theoretical Calculation of Phase Transition in Ru-Doped MoS_2_

**Figure 1:**
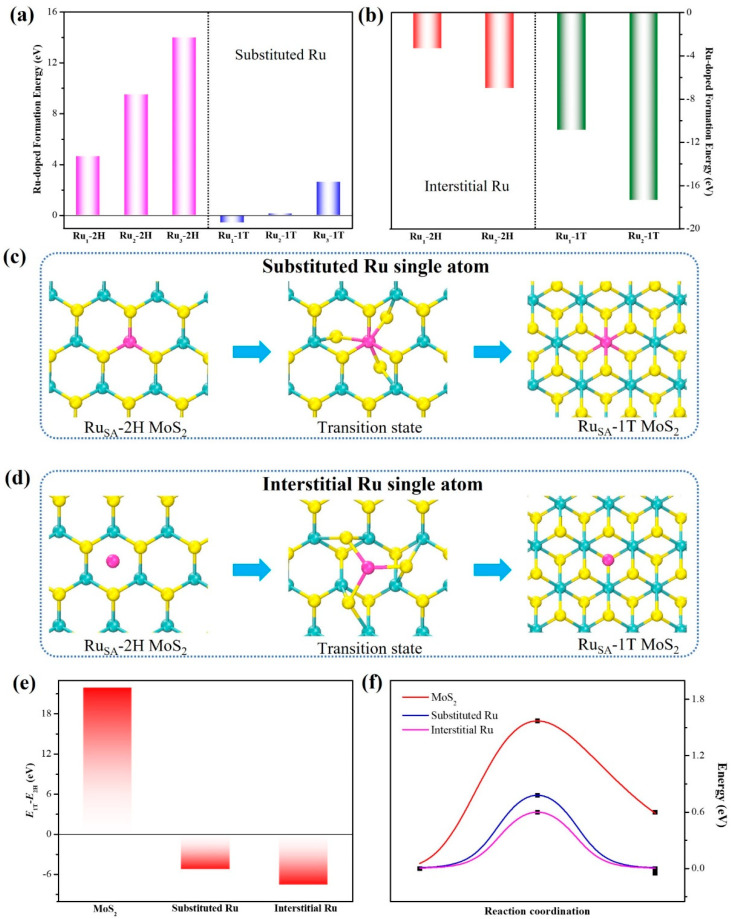
The formation energy of Ru substitutional doping (**a**) and Ru interstitial doping (**b**) in the 2H and 1T phases of MoS_2_. Illustrative schematic depicting the evolutionary process of crystal structures for substitutional doping of single Ru atoms (**c**) and for interstitial doping of single Ru atoms (**d**) in the 2H-to-1T transition. (**e**) The difference in formation energy for 2H-to-1T transition of MoS_2_, substitutional Ru-doped MoS_2_, and interstitial Ru-doped MoS_2_. (**f**) Phase transition barriers of MoS_2_, substitutional Ru-doped MoS_2_, and interstitial Ru-doped MoS_2_.

The theoretical calculation about the influence of ruthenium doping content and location on the 2H to 1T transition was discussed firstly. Figure 1a illustrated the substitutional doping of Ru atoms with different content in 2H and 1T phases. The results suggested that it was favorable to dope Ru at substitutional sites in 1T than in 2H MoS_2_. The lowest formation energy is about −0.570 eV, especially for the single-atom ruthenium doping. To facilitate a more accurate comparison of various initial doping positions, the formation energies for interstitial sites of Ru doping have also been calculated. As depicted in Figure 1b, the negative formation energies indicate that the Ru doping of interstitial sites is more energetically favorable than the substitutional sites. Figure 1c,d show the structural evolution process of Ru doping in 2H as viewed from the top. The 2H-to-1T transition process was noted when Ru atoms were introduced into 2H MoS_2_ and formed Ru-S bonds with surrounding sulfur. To facilitate a more accurate comparison of the phase transition, the difference in formation energy for 2H-to-1T transition (∆*E* = *E*_2H_ − *E*_1T_) has been calculated [44]. As shown in Figure 1e, the ∆*E* values changed from −7.55 to 21.95 eV, indicating that the phase transition was thermodynamically more favorable for Ru doping at interstitial sites than at substituted sites. The 2H-to-1T transition process, initiating with the rotation of chalcogen atoms adjacent to the Ru atom and culminating in the stabilization of the structure, was shown in Figure 1c,d. The kinetic barriers for the phase transitions in MoS_2_, substitutional Ru-doped MoS_2_, and interstitial Ru-doped MoS_2_, were calculated using the following equation [5].

*E*_barrier_ = *E*_highest_ − *E*_initial_ (7)

Figure 1f shows the energy variations. The calculated values for the kinetic barrier, *E*_barrier_, were 1.57 eV for MoS_2_, 0.78 eV for substitutional Ru-doped MoS_2_, and 0.60 eV for interstitial Ru-doped MoS_2_, respectively. In comparison to pure MoS_2_, the significantly reduced energy barrier for phase transition in substitutional Ru-doped MoS_2_ and interstitial Ru-doped MoS_2_ indicated that Ru atoms played a key role in 2H-to-1T transition. Thus, the 2H-to-1T transition from the semiconducting to metallic polymorph through Ru doping occurs spontaneously.

### 3.2. Synthesis and Characterization

**Figure 2:**
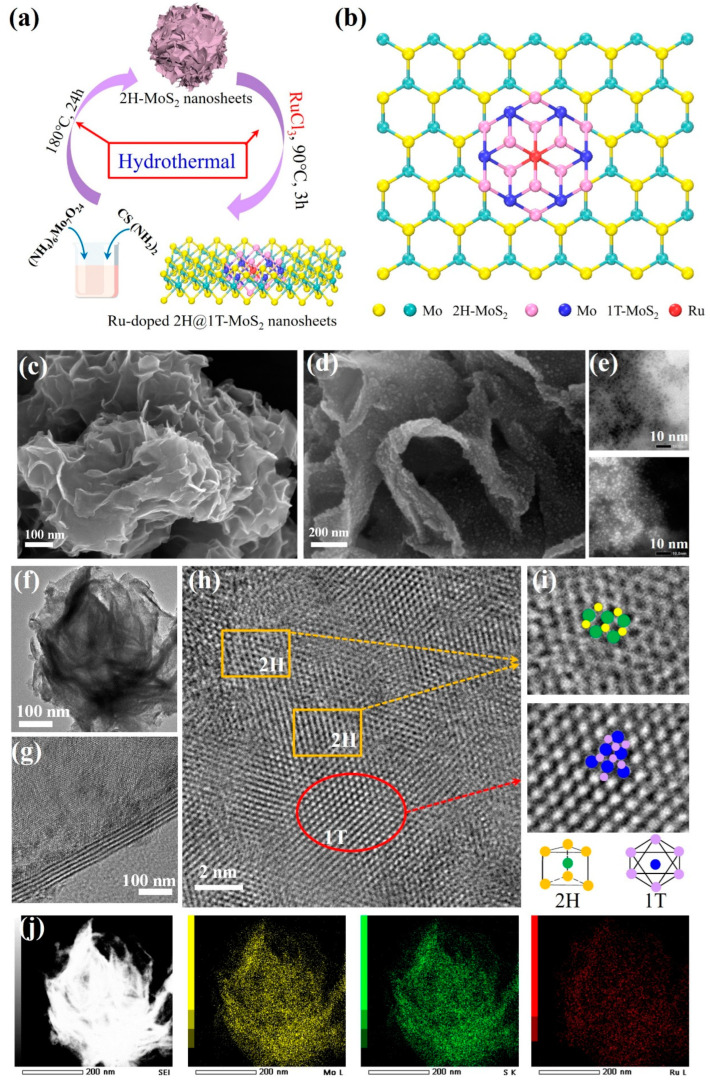
(**a**) Schematic diagram of the two-step hydrothermal method of Ru-doped MoS_2_ nanosheets. (**b**) Depiction of Ru-doped MoS_2_ with 2H-to-1T transition. SEM images of MoS_2_ (**c**) and Ru-doped MoS_2_ (**d**). (**e**) TEM bright-field (**up**) and dark-field (**down**) images of Ru-doped MoS_2_. TEM and HRTEM images of Ru-doped MoS_2_ (**f**–**i**). (**j**) Dark-field TEM images and elemental mapping of Ru-doped MoS_2_.

Using a conventional two-step hydrothermal method, 2H-phase MoS_2_ nanosheets were employed as the substrate to produce the Ru-doped MoS_2_ with varying Ru doping concentrations, as illustrated in Figure 2a. Consistent with the results of DFT calculation, the incorporation of Ru atoms could facilitate the formation of stable structures through bonding with sulfur, and promote the rotation of chalcogen atoms in the vicinity of the Ru (Figure 2b). To investigate the morphology and structure of the as-prepared materials, FESEM and TEM were employed. Pure MoS_2_ showed the typical 2D ultrathin nanosheets, which agglomerated vertically to form porous microspheres as depicted in Figure 2c and Figure S1. Additionally, the pure MoS_2_ exhibited smooth surfaces. Ru-doped MoS_2_ showed a similar structural profile to pure MoS_2_, and the nanosheets displayed no evident layer stacking (Figures S2 and S3). The results suggested that the Ru incorporation had little influence on the intrinsic morphology of MoS_2_. It could see the distinct structural features in Figure 2d. Ru-doped MoS_2_ exhibited the coarse surfaces with numerous nanoparticles, which would enhance the surface area of MoS_2_. To investigate the nanoparticles on the surface, TEM bright-field and dark-field imaging techniques were employed, as shown in Figure 2e. The bright-field TEM revealed a multitude of black dots, which transformed into light dots under dark-field conditions. The results suggested the successful incorporation of Ru atoms on the MoS_2_ surfaces. However, the XRD patterns of pure MoS_2_ and Ru-doped MoS_2_ only exhibited peaks that are assigned to the standard diffraction patterns of MoS_2_ (PDF#37-1492) in Figure S4. These peaks were observed at 13.9°, 33.3°, 39.7°, and 58.7°, which correspond to the (002), (100), (103), and (110) lattice planes, respectively. Furthermore, no distinct diffraction peaks of Ru-based compounds were detected in the XRD patterns due to the low doping concentration, the amorphous nature, or the uniform dispersion of Ru within MoS_2_. TEM images confirmed that both pure MoS_2_ and Ru-doped MoS_2_ exhibited rose-like architecture composed of delicate and translucent nanoflakes as depicted in Figure 2f and Figure S5. The magnified TEM image showed a lattice fringe with a spacing of ~0.68 nm, which corresponds to the (002) planes of MoS_2_, as illustrated in Figure 2g [36,37]. A distinction in lattice structure between 2H and 1T phases of MoS_2_ was observed in Ru-doped MoS_2_ (Figure 2h), indicating the coexistence of both phases within the materials. Moreover, the HRTEM images of MoS_2_ revealed the presence of the trigonal lattice characteristic of the 1T phase, in addition to the prevalent honeycomb lattice areas typical of the trigonal prismatic coordination found in the 2H phase (Figure 2i). EDX further confirmed the presence of Mo, S, and Ru elements (Figure 2j). These results confirmed the successful fabrication of Ru-doped MoS_2_ electrocatalysts, and the introduction of Ru resulted in the phase incorporation of 2H@1T MoS_2_.

**Figure 3:**
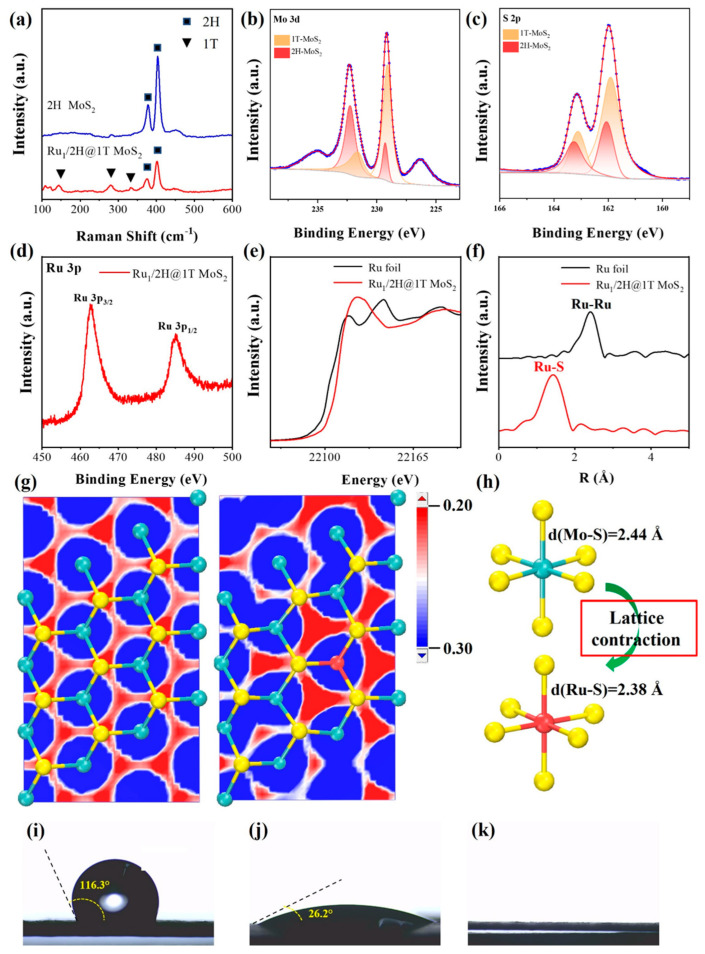
(**a**) Raman spectra of MoS_2_ and Ru-doped MoS_2_. X-ray photoelectron spectroscopy of the Mo 3d orbitals (**b**) and the S 2p orbitals (**c**) of Ru-doped MoS_2_. (**d**) X-ray photoelectron spectroscopy of the Ru 3p orbitals of Ru-doped MoS_2_. (**e**) Normalized Ru K-edge XANES spectra of Ru-doped MoS_2_ and Ru foil. (**f**) k^2^-weighted EXAFS spectra of Ru-doped MoS_2_ and Ru foil. (**g**) The difference of electron density for MoS_2_ and Ru-doped MoS_2_. (**h**) Structural change model of MoS_2_ after Ru doping. Contact angle measurements of carbon paper (**i**), Ru-doped MoS_2_ catalysts (**j**), and Ru-doped MoS_2_ catalysts on carbon paper (**k**).

In order to further demonstrate MoS_2_ transition from 2H to 1T phases, Raman spectroscopy was employed to support the direct evidence. A crucial difference between 2H-MoS_2_ and 1T-MoS_2_ is the symmetry of the S atoms in their structures, which causes an obvious difference in Raman spectral characteristics. As shown in Figure 3a, the 2H MoS_2_ materials only displayed two bands at 383 and 408 cm^−1^. These two peaks are assignable to the in-plane Mo-S phonon mode (E_2g_) and the out-of-plane Mo-S phonon mode (A_1g_), respectively [45]. For Ru-doped MoS_2_, there were three new bands at 156, 226, and 330 cm^−1^ observed, corresponding to *J*_1_, *J*_2_, and *J*_3_, respectively. These three bands are corresponded to the phonon modes of 1T-MoS_2_, thus confirming the 2H-to-1T transition [6,36]. In order to study the electronic effects of Ru-doped MoS_2_, XPS analysis was employed. In contrast with 2H-MoS_2_ (Figures S6 and S7), both Mo 3d and S 2p XPS spectra of 0.08-Ru/2H@1T MoS_2_ showed a shift of 0.50 (±0.05) eV toward low binding energy. The spectra of Mo 3d and S 2p were fitted, and they were deconvoluted into four peaks. For Mo 3d spectra, two weak peaks, corresponding to the 3d_5/2_ and 3d_3/2_ of Mo^4+^ species from the 2H phase, located at 229.3 and 232.3 eV, as well as two peaks observed at 229.1 and 231.6 eV, which revealed the presence of the 1T phase [45,46]. Figure 3c reveals that the S 2p spectra of 0.08-Ru/2H@1T MoS_2_ and 2H-MoS_2_ were also fitted into four peaks. For S 2p spectra, there were two weak peaks, corresponding to the 2p_3/2_ and 2p_1/2_ of S^2−^ species from the 2H phase, located at 162.1 and 163.3 eV, and two peaks observed at 161.9 and 163.0 eV proved the presence of the 1T phase [47]. Figure 3d exhibited two distinct characteristic peaks at 462.5 eV and 485.0 eV, respectively, attributed to the 3p_3/2_ and 3p_1/2_ orbitals of Ru. Notably, no peak was observed at the 280.2 eV position, which is characteristic of zero-valence Ru [48], which suggested that Ru atoms would be coordinated with S atoms.

A contrast of XANES spectra between Ru foil and Ru-doped MoS_2_ was depicted in Figure 3e. It was noted that the differences included the variations in transition strength and the displacement in the absorption edge, which correspond to the 1s to 5p electronic transition. The Ru K-edge energy of Ru-MoS_2_ was observed to shift to a higher energy level, ranging between 22,150 and 22,200 eV, which indicated that Ru atoms possessed a more positive charge in Ru-doped MoS_2_ [49,50,51]. The results further proved that Ru was predominantly bonded to S atoms of MoS_2_. The extent XAFS was further deployed to investigate the fine structure of Ru-doped MoS_2_ [52,53]. The FT *R*-space spectra in Figure 3f illustrated that Ru-doped MoS_2_ lacked a scattering peak at approximately 2.37 Å, which is a prominent feature in the spectrum of the Ru foil [54]. The results indicated the absence of Ru-Ru bonding in the Ru-doped MoS_2_. Furthermore, there was only the first-shell scattering peak at around 1.44 Å in the Ru-doped MoS_2_, which corresponds to the Ru-S bonds. The results were in agreement with the XPS results. Figure 3g showed the local structures of Mo-S and Ru-S in both MoS_2_ and Ru-doped MoS_2_, along with the electron density differences. The excess electrons at the S sites tended to migrate towards the Ru atoms after the introduction of Ru, leading to the increased electron density around Ru and the electron depletion at the adjacent S sites [28,55]. The results are in agreement with XPS, XANES and DFT calculations. Additionally, it can be seen from Figure 3h that the Ru introduction altered the local twisted structures within the MoS_2_ lattice, which would create the additional active sites for H* adsorption. Meanwhile, the contact angle between water and the Ru-doped MoS_2_/CP electrocatalysts exhibits an expected reduction when contrasted with CP and the Ru-doped MoS_2_ catalyst, attributable to the enhanced hydrophilicity of the synergy effect (Figure 3i–k).

### 3.3. Electrocatalytic H_2_ Evolution and Electrochemical Performances

**Figure 4:**
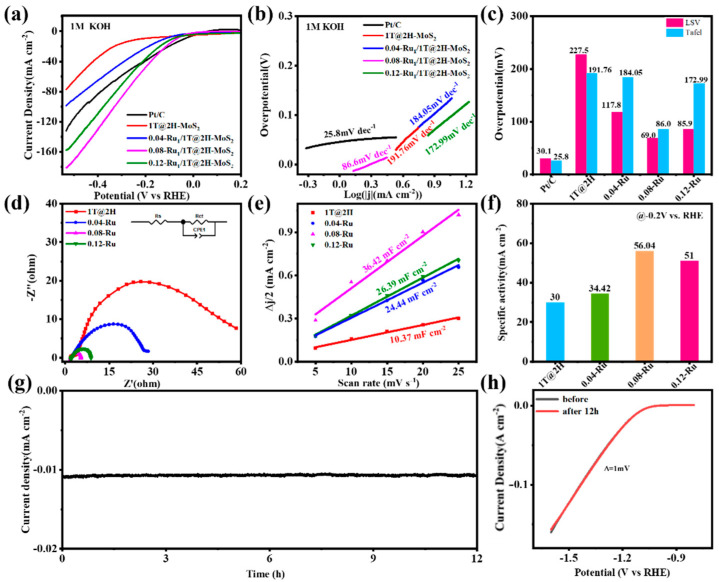
(**a**) LSV of MoS_2_ and Ru-doped MoS_2_ samples in 1 M KOH solution at a scan rate of 5 mV/s. (**b**) Tafel plots derived from the polarization curves for all samples. (**c**) Overpotentials and Tafel slopes at 10 mA cm^−2^. (**d**) EIS of MoS_2_ and Ru-doped MoS_2_ samples. (**e**) The electrochemical double-layer capacitance. (**f**) The specific activity of MoS_2_ and Ru-doped MoS_2_ samples. (**g**) Chronoamperometric stability profiles over time during the HER test recorded for 12 h. (**h**) Polarization curves before and after 1000 CV cycles.

The evaluation of the catalysts’ electrocatalytic HER performance was performed in 1 M KOH electrolyte firstly. As depicted in the linear sweep voltammetry curves (without IR compensation), 0.08-Ru/1T@2H-MoS_2_ exhibited superior HER performance compared to 0.04-Ru/1T@2H-MoS_2_, 0.12-Ru/1T@2H-MoS_2_, and MoS_2_ composite (Figure 4a). The 0.08-Ru/1T@2H-MoS_2_ exhibited high HER activity in the range of −50 to −170 mA cm^−2^, which was better than the commercial 20 wt% Pt/C catalyst. Furthermore, the Tafel slope for 0.08-Ru/1T@2H-MoS_2_ was 86 mV dec^−1^, significantly lower than the 191.7 mV dec^−1^ observed for MoS_2_ composite, 184 mV dec^−1^ for 0.04-Ru/1T@2H-MoS_2_, and 172 mV dec^−1^ for 0.12-Ru/1T@2H-MoS_2_ (Figure 4b). Additionally, Figure 4c illustrated a comparison of the overpotentials and Tafel slopes required at the current density of 10 mA cm^−2^ (*η* 10). The overpotential for 0.08-Ru/1T@2H-MoS_2_ is only 61 mV, which further demonstrated that the catalytic process followed the Volmer–Heyrovsky mechanism [56]. As depicted in Figure 4d, the kinetics of charge transfer for the catalysts was probed using electrochemical impedance spectroscopy (EIS) at the overpotential of 100 mV. The calculated charge transfer resistances (*R*_ct_) were 4.2, 7.1, 24.8, and 59.3 Ω for 0.08-Ru/1T@2H-MoS_2_, 0.12-Ru/1T@2H-MoS_2_, 0.04-Ru/1T@2H-MoS_2_, and MoS_2_ composite. The accelerated electron transfer in 0.08-Ru/1T@2H-MoS_2_, as compared to MoS_2_ composite, was predominantly due to the introduction of Ru atoms. The results indicated that the introduction of Ru not only leaded to the phase transition of MoS_2_ but improved the electron transfer from electrocatalysts to solution for hydrogen evolution. As depicted in Figure 4e, the double-layer capacitance (*C*_dl_) was determined from cyclic voltammetry (CV) curves (Figure S8). The 0.08-Ru/1T@2H-MoS_2_ exhibited a *C*_dl_ value of 36.42 mF cm^−2^—more than 0.12-Ru/1T@2H-MoS_2_ (26.39 mF cm^−2^), 0.04-Ru/1T@2H-MoS_2_ (24.22 mF cm^−2^), and MoS_2_ composite (10.37 mF cm^−2^). These results demonstrated that 0.08-Ru/1T@2H-MoS_2_ possessed a substantial electrochemical active surface area (ECSA), which provided a greater number of catalytic active sites. As shown in Figure 4f, the specific activity of 0.08-Ru/1T@2H-MoS_2_ was more than double that of the MoS_2_ composite. Figure 4g,h illustrated the evaluation of the electrochemical stability of 0.08-Ru/1T@2H-MoS_2_ through a 12 h chronoamperometric test, exhibiting the superior operational durability and chemical stability. Furthermore, the catalyst maintained no significant change in overpotential when compared to the initial measurement of the LSV curve after 12 h of electrochemical stability testing.

**Figure 5:**
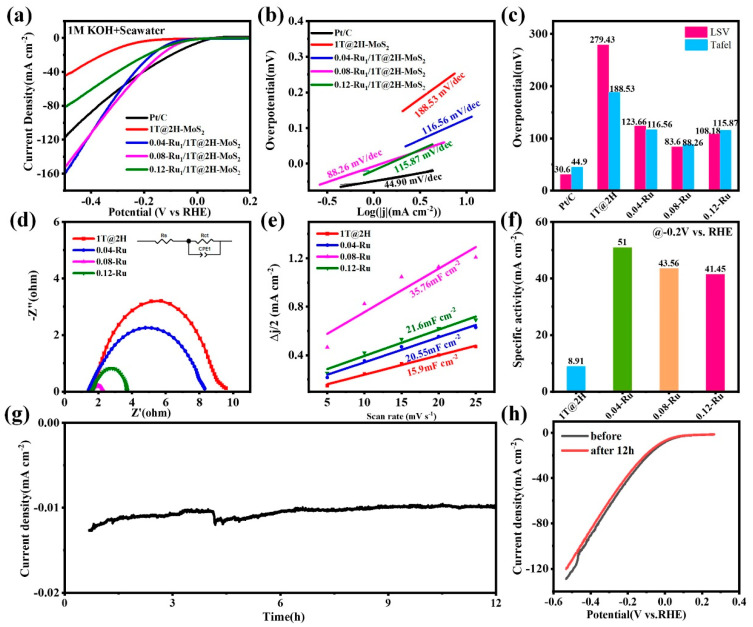
(**a**) LSV of MoS_2_ and Ru-doped MoS_2_ samples in alkaline seawater (1 mol KOH dissolved in 1 L seawater) at a scan rate of 5 mV/s. (**b**) Tafel plots derived from the polarization curves for all samples. (**c**) Overpotentials and Tafel slopes at 10 mA cm^−2^. (**d**) EIS of MoS_2_ and Ru-doped MoS_2_ samples. (**e**) The electrochemical double-layer capacitance. (**f**) The specific activity of MoS_2_ and Ru-doped MoS_2_ samples. (**g**) Chronoamperometric stability profiles over 12 h during the HER test. (**h**) Polarization curves before and after 1000 CV cycles.

Given the outstanding catalytic activity in the 1 M KOH solution, the HER capabilities of the 0.08-Ru/1T@2H-MoS_2_ catalysts were measured in alkaline seawater (1 mol KOH dissolved in 1 L seawater). As depicted in Figure 5a–c, the overpotential required for 0.08-Ru/1T@2H-MoS_2_ to achieve a current density of 10 mA cm^−2^ was only 83 mV, and its Tafel slope was 88 mV dec^−1^. The results indicated that 0.08-Ru/1T@2H-MoS_2_ also possessed efficient electrocatalytic HER performance in alkaline seawater, and consistently followed the Volmer–Heyrovsky reaction mechanism. A comparison of hydrogen evolution reaction performance for MoS_2_ and Ru-doped MoS_2_ with other catalysts in the previous reports was summarized in Table S1. Furthermore, the electrochemical impedance spectroscopy results demonstrated that 0.08-Ru/1T@2H-MoS_2_ still exhibited a lower charge transfer resistance of 0.8 Ω, compared to 0.12-Ru/1T@2H-MoS_2_ (2.2 Ω), 0.04-Ru/1T@2H-MoS_2_ (6.3 Ω), and MoS_2_ composite (8.1 Ω), respectively (Figure 5d). Notably, the *R*_ct_ values for these samples in alkaline seawater were lower compared to those in a 1M KOH solution. The enhanced conductivity of the catalysts in seawater could be attributed to the higher concentration of ionic impurities, predominantly sodium chloride (Na^+^, Cl^−^) and other salt ions present in the seawater. As displayed in Figure 5e, the double-layer capacitance (*C*_dl_) was determined from cyclic voltammetry (CV) curves (Figure S9). The 0.08-Ru/1T@2H-MoS_2_ had the highest *C*_dl_ value (35.8 mF cm^−2^) among the tested samples. The *C*_dl_ of 0.12-Ru/1T@2H-MoS_2_, 0.04-Ru/1T@2H-MoS_2_, and MoS_2_ composite were 21.6 mF cm^−2^, 20.6 mF cm^−2^, and 15.9 mF cm^−2^, respectively. The specific activity of 0.08-Ru/1T@2H-MoS_2_ exceeded twice that of MoS_2_ composite (Figure 5f). Drawing from the ECSA and specific activity results in alkaline seawater, it could be concluded that the intrinsic activity of the catalysts was superior in a 1M KOH solution. Consequently, it could be inferred that the blocking of active sites by diverse microorganisms, along with the enrichment of chloride ions and the corrosive action of alkaline earth cations (Ca^2+^ and Mg^2+^), contribute to the degradation of the catalysts [57]. Figure 5g,h displayed the outcomes of stability tests conducted in alkaline seawater. The durability of 0.08-Ru/1T@2H-MoS_2_ showed mild fluctuations, and there was a slight increase in the overpotential of 0.08-Ru/1T@2H-MoS_2_ following the 12 h stability test. In comparison to the 1 M KOH solution, the reduced stability of the catalysts in alkaline seawater was attributed to the hypochlorite and chlorine produced by the chlorine evolution reaction (CER), which competed with the oxygen evolution reaction (OER) at the anode. These species could impede the hydrogen evolution reaction and cause electrode corrosion [58]. In conclusion, the intricate composition of alkaline seawater leaded to only a negligible decline in the electrochemical performance of the materials.

### 3.4. Mechanism

**Figure 6:**
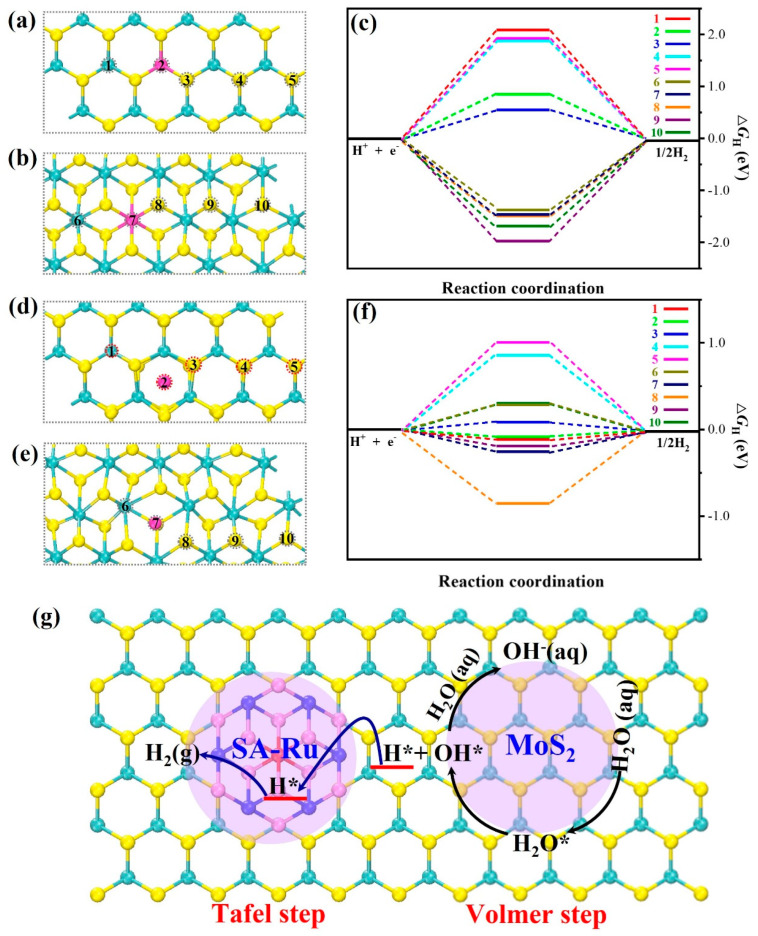
Top views of H atom adsorbing on substitutional Ru-doped 2H MoS_2_ (**a**) and Ru-doped 1T MoS_2_ (**b**). (**c**) The computed free energy landscapes for HER on substitutional Ru-doped MoS_2_ surfaces. Top views of H atom adsorbing on interstitial Ru-doped 2H MoS_2_ (**d**) and Ru-doped 1T MoS_2_ (**e**). (**f**) The computed free energy landscapes for HER on interstitial Ru-doped MoS_2_ surfaces. (**g**) Proposed mechanism for HER on Ru-doped MoS_2_ catalysts.

To gain insight into the actual active sites of Ru-doped MoS_2_, the Gibbs free energies (∆*G*_H*_) of hydrogen adsorption at various active sites were calculated. Typically, an efficient catalyst exhibits an exceptional ability to adsorb and desorb hydrogen at edge sites (∆*G*_H*_ close to 0 eV). For the substitutional Ru-doped MoS_2_ catalysts, ten distinct sites were chosen as potential hydrogen adsorption locations, as illustrated in Figure 6a,b. Figure 6c presented the free energy diagram for substitutional Ru-doped MoS_2_ at all active sites involving Mo-S and Ru-S bonds. The analysis revealed that hydrogen adsorption at the 2H Mo-S (Site 1) and 1T Mo-S (Site 9) terminations was excessively strong. The ∆*G*_H*_ values were too high, indicating that it was difficult to adsorb hydrogen on the substitutional Ru-doped MoS_2_ catalysts. For the interstitial Ru-doped MoS_2_ catalysts, ten distinct sites were also identified as potential hydrogen adsorption sites, as depicted in Figure 6d,e. Figure 6f presented the free energy diagram for the interstitial Ru-doped MoS_2_. There were so many sites with optical hydrogen adsorption energies close to zero, 2H Ru-S (Site 2: 0.085 eV) and Mo-S (Site 3: −0.083 eV). The results suggested that these two sites were more optimal for hydrogen adsorption and desorption. Moreover, the H adsorption on the interstitial Ru-doped MoS_2_ catalysts was more advantageous for the HER compared to the adsorption on the substitutional Ru-doped MoS_2_ catalysts. Based on the above results, a proposed reaction mechanism for the alkaline HER on Ru-doped MoS_2_ catalysts was depicted in Figure 6g. In this mechanism, the interstitial Ru sites within the model exhibited the enhanced activity during the Volmer step, efficiently facilitating the catalytic dissociation of water. Subsequently, the hydrogen intermediates (H*) formed on the Ru sites were then transferred to the S sites in the 2H phase of MoS_2_ via a spillover process. At these S sites, the catalyst showed superior activity during the Tafel step, promptly combining the transferred H* to produce H_2_. This process effectively prevented the deactivation of the active sites involved in water dissociation, which could occur due to the accumulation of H*. In summary, the remarkable alkaline HER performance of Ru-doped MoS_2_, particularly at high ampere-level current densities, could be attributed to the efficient hydrogen spillover phenomenon occurring on the Ru-doped MoS_2_.

## 4. Conclusions

In conclusion, the enhancement of the hydrogen evolution reaction performance in Ru-doped MoS_2_ nanosheets is achieved through a synergistic tuning of structure and conductivity via Ru doping. This process spontaneously formed a 2H@1T MoS_2_ heterojunction, leading to a redistribution of charges between Ru and MoS_2_. The unique structure acted as a catalyst for the rapid hydrogen spillover on Ru sites, where Ru facilitated water dissociation, while the modified S sites managed efficient hydrogen adsorption and desorption, as confirmed by DFT and experimental results. This hydrogen spillover, initiated by the engineered Ru atoms, endowed Ru-doped MoS_2_ with the capability to operate stably at industrial ampere-level current densities under ultra-low overpotentials in alkaline environments. Consequently, the Ru-doped MoS_2_ catalyst achieved the overpotentials of 61 mV at 10 mA cm^−2^ in 1.0 M KOH, 79 mV in alkaline seawater, respectively. This work not only provides design concepts and fabrication strategies for catalysts, but also highlights the vast potential of hydrogen spillover strategy in constructing catalysts for high-current-density hydrogen evolution.

## Data Availability

Data are contained within the article and Supplementary Materials.

## Supplementary Materials

The following supporting information can be downloaded at https://www.mdpi.com/article/10.3390/nano15100777/s1, Figure S1: SEM images of MoS2; Figure S2: SEM images of 0.04-Ru/1T@2H-MoS2 (a), 0.08-Ru/1T@2H-MoS2 (b), and 0.12-Ru/1T@2H-MoS2 (c); Figure S3: SEM images of 0.04-Ru/1T@2H-MoS2 (a), 0.08-Ru/1T@2H-MoS2 (b), and 0.12-Ru/1T@2H-MoS2 (c); Figure S4: XRD patterns of MoS2 and Ru-doped MoS2; Figure S5: TEM images of MoS2 (a) and 0.08-Ru/1T@2H-MoS2 (b); Figure S6: X-ray photoelectron spectroscopy of the Mo 3d orbitals of MoS2; Figure S7: X-ray photoelectron spectroscopy of the S 2p orbitals of MoS2; Figure S8: Cyclic voltammetry of MoS2 (a), 0.04-Ru/1T@2H-MoS2 (b), 0.08-Ru/1T@2H-MoS2 (c), and 0.12-Ru/1T@2H-MoS2 (d) in 1.0 M KOH solution; Figure S9: Cyclic voltammetry of MoS2 (a), 0.04-Ru/1T@2H-MoS2 (b), 0.08-Ru/1T@2H-MoS2 (c), and 0.12-Ru/1T@2H-MoS2 (d) in alkaline seawater; Table S1: Comparison of hydrogen evolution reaction performance for MoS_2_ and Ru-doped MoS_2_ with other catalysts. Refs. [37,59,60,61,62,63,64,65,66,67,68] are cited in the Supplementary Materials.
