# Supplementary material for: Ru-Doped Induced Phase Engineering of MoS2 for Boosting Electrocatalytic Hydrogen Evolution
Source: Nanomaterials (Basel). 2025 May 21;15(10):777. doi: 10.3390/nano15100777 (PMC12114032; doi:10.3390/nano15100777)
Supplement: Supplementary file 1 [file nanomaterials-15-00777-s001.zip › nanomaterials-3629630-supplementary.pdf]

Supplementary Material

Ru-Doped Induced Phase Engineering of MoS_2 for Boosting Electrocatalytic Hydrogen Evolution

njie Li †, Meng Yu †, Junjie Li, Ning Wang, Xiaolong Yang and Yanhua Peng*

College of Chemistry and Chemical Engineering, Qingdao University, Qingdao 266071, China.

*Corresponding Authors

Email: yhpeng@qdu.edu.cn

† These authors contributed equally to this work.

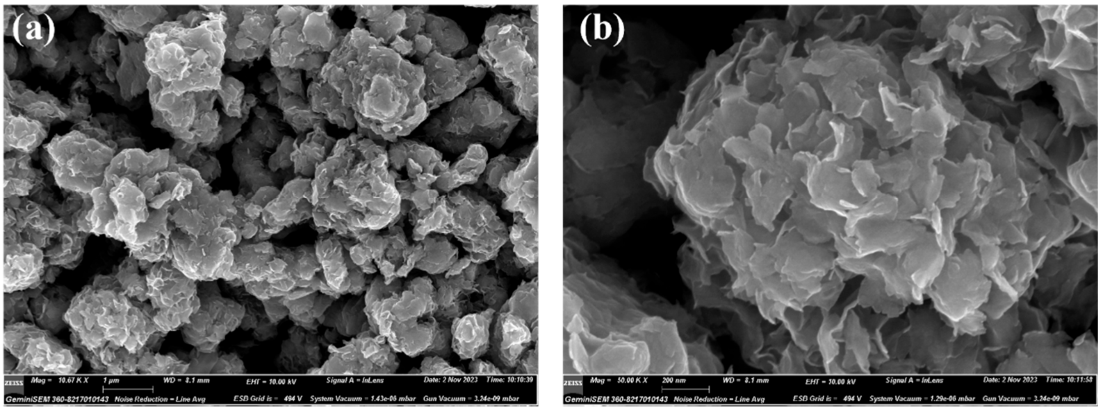

Fig. S1. SEM images of MoS_2.

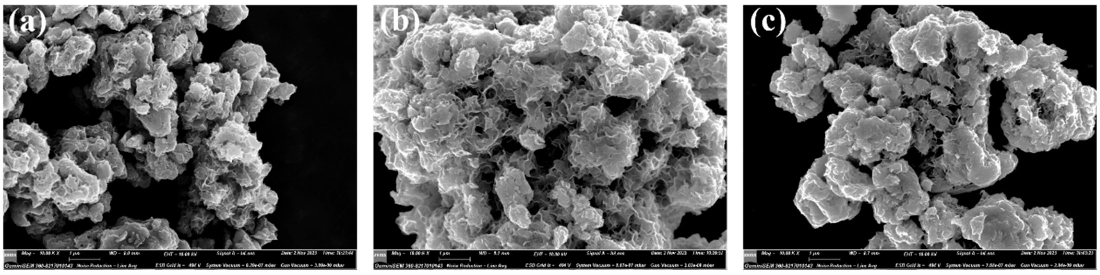

Fig. S2. SEM images of 0.04-Ru/1T@2H-MoS_2 (a), 0.08-Ru/1T@2H-MoS_2 (b) and 0.12-Ru/1T@2H-MoS_2 (c).

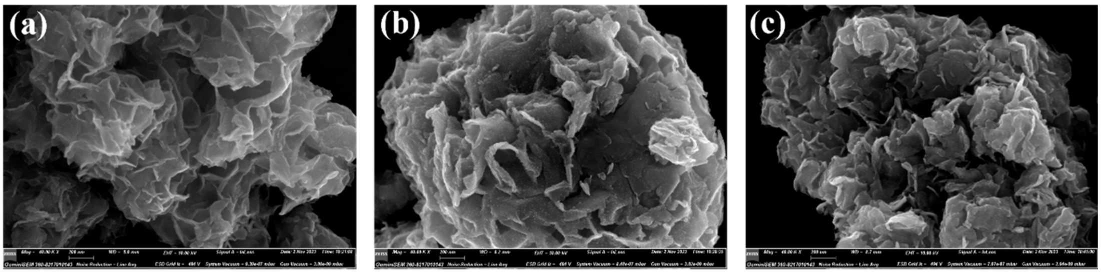

Fig. S3. SEM images of 0.04-Ru/1T@2H-MoS_2 (a), 0.08-Ru/1T@2H-MoS_2 (b) and 0.12-Ru/1T@2H-MoS_2 (c).

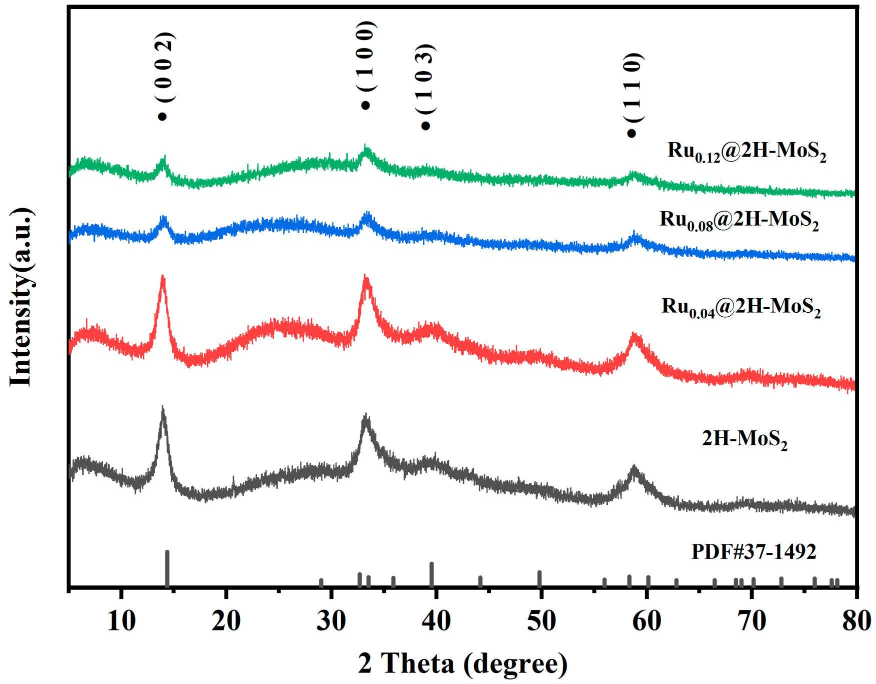

Fig. S4. XRD patterns of MoS_2 and Ru-doped MoS_2.

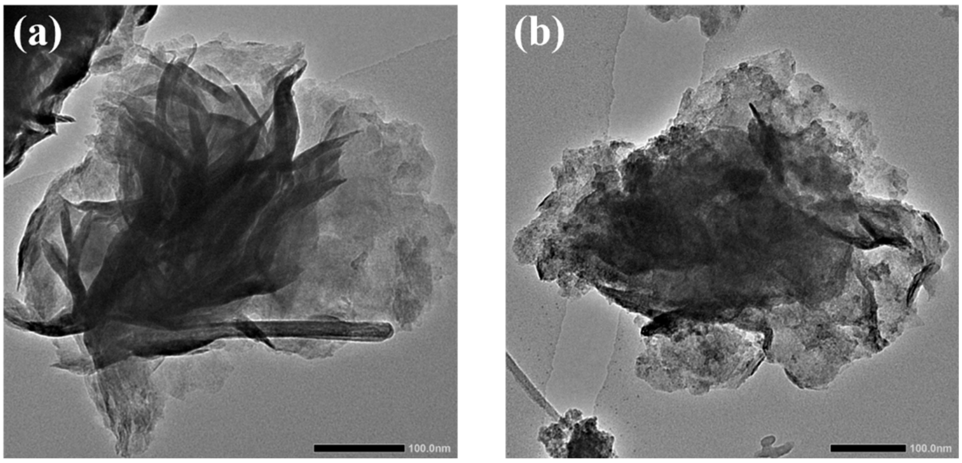

Fig. S5. TEM images of MoS_2 (a) and 0.08-Ru/1T@2H-MoS_2 (b).

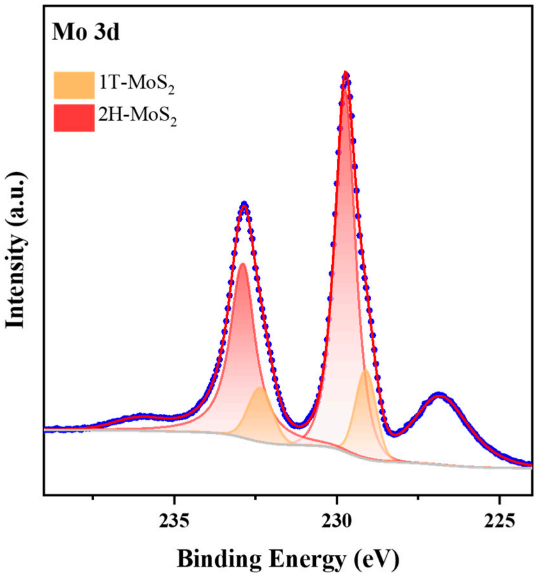

Fig. S6. X-ray photoelectron spectroscopy of the Mo 3d orbitals of MoS_2.

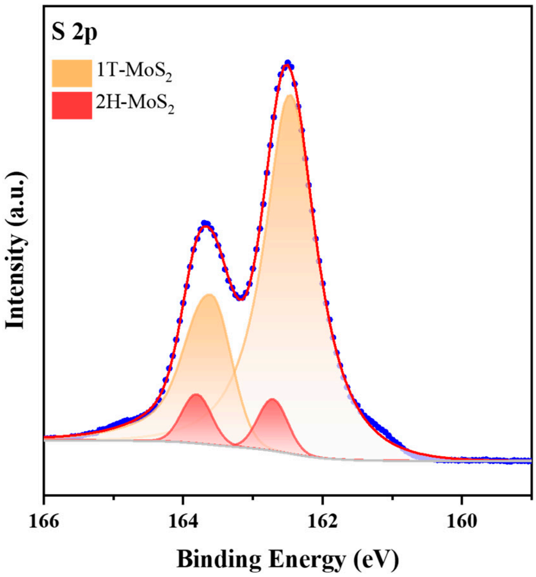

Fig. S7. X-ray photoelectron spectroscopy of the S 2p orbitals of MoS_2.

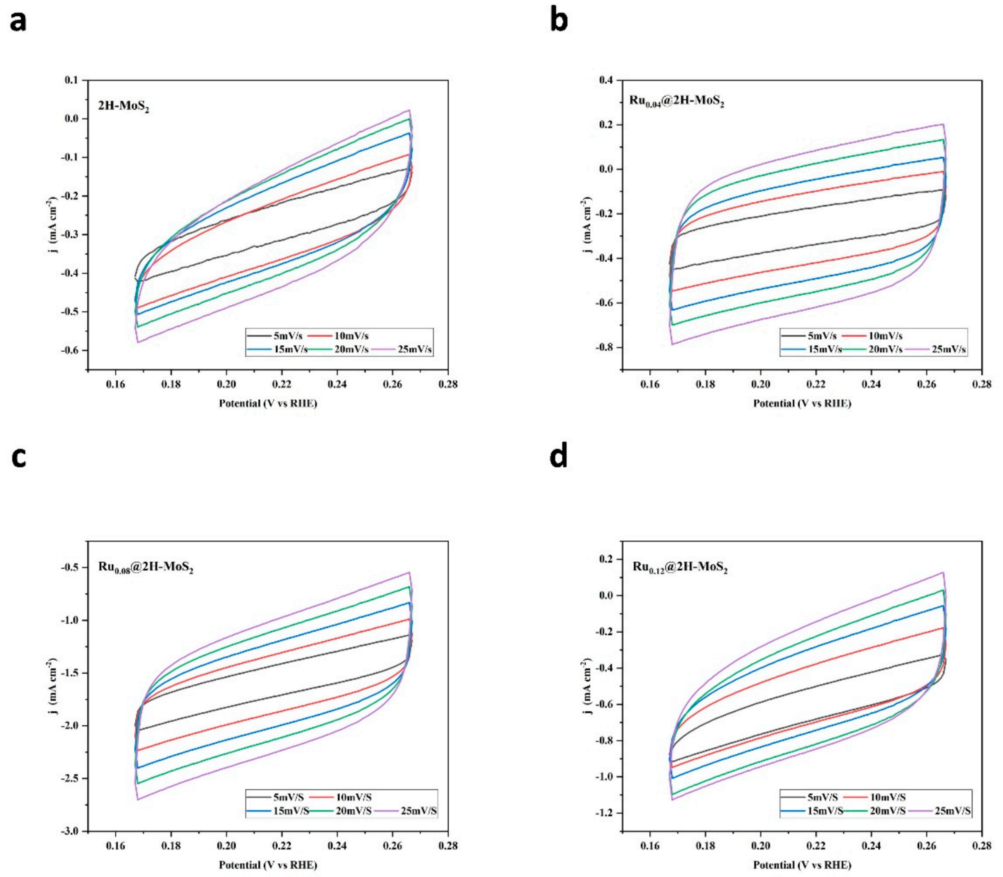

Fig. S8. Cyclic voltammetry of MoS_2 (a), 0.04-Ru/1T@2H-MoS_2 (b), 0.08-Ru/1T@2H-MoS_2 (c) and 0.12-Ru/1T@2H-MoS_2 (d) in 1.0 M KOH solution.

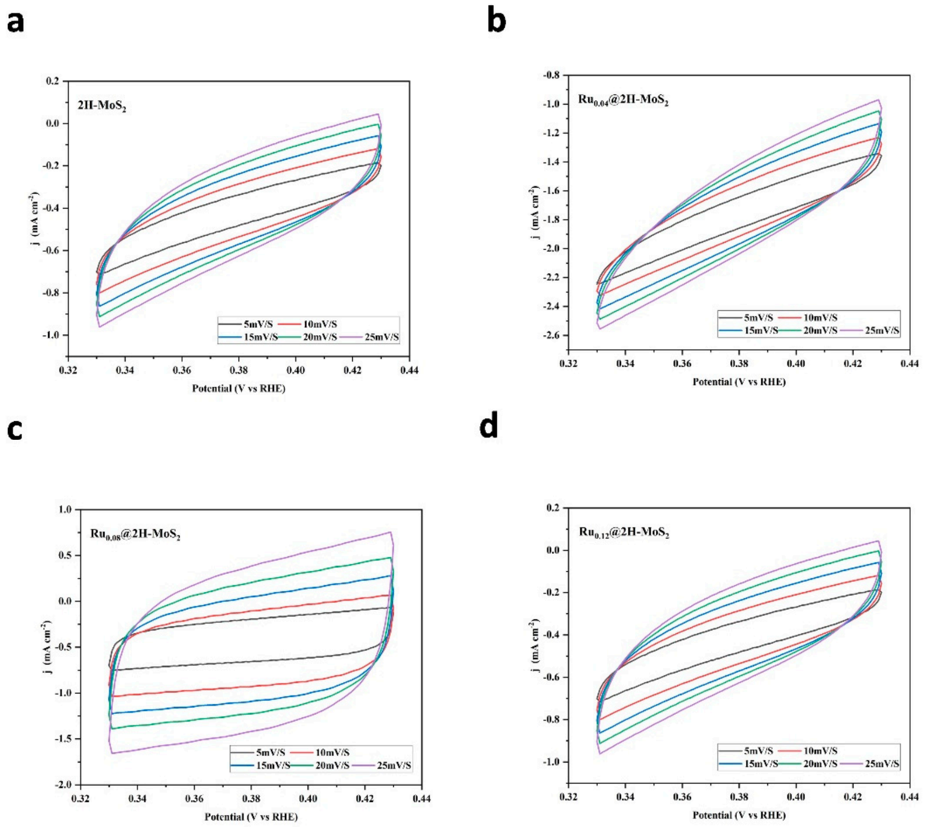

Fig. S9. Cyclic voltammetry of MoS_2 (a), 0.04-Ru/1T@2H-MoS_2 (b), 0.08-Ru/1T@2H-MoS_2 (c) and 0.12-Ru/1T@2H-MoS_2 (d) in alkaline seawater.

Table S1. Comparison of hydrogen evolution reaction performance for MoS_2 and Ru-doped MoS_2 with other catalysts.

Sample	HER η_{10} (mV) / Tafel Slope (mV dec^{-1})		Ref.
	Alkaline seawater	1.0 KOH solution	
MoS_2	282/202	309/191	This work
Ru-doped 1T@2H-MoS_2	83/88	69/86	This work
Ru0.10@2H-MoS2	/	51/64.9	[1]
1T-2H MoS_2/CoS_2	/	37/46	[2]
Co-MoS_2/V_2C@CC	/	70.1/98.6	[3]
Ni-1T-MoS_2	/	199/52.7	[4]
Cu_{sub}@MoS_2	160/86	/	[5]
Pt–Ni@NiMoN/NF	11/30.6	/	[6]
Ni−Co@Fe−CoPBA	183/60	43/53	[7]
Cr-Co_XP	/	100/75.6	[8]
SA-MoO_2/$Ni_3(PO_4)_2$	46/62	34/43.9	[9]
Pd-MS5	/	149/72	[10]
Ru SAs-NiP	/	57/75	[11]

Reference

[1] J. Wang, W. H. Fang, Y. Hu, Y. H. Zhang, J. Q. Dang, Y. Wu, B. Z. Chen, H. Zhao, Z. X. Li, Single atom Ru doping 2H-MoS_2 as highly efficient hydrogen evolution reaction electrocatalyst in a wide pH range. Appl. Catal. B: Environ. 298 (2021)120490.

[2] P. Chang, T. Wang, Z. Liu, X. Wang, J. Zhang, H. Xiao, L. Guan, J. Tao, Interface-assisted phase transition in MOF-Derived MoS_2/CoS_2 heterostructures for highly efficient Dual-pH hydrogen evolution and overall water splitting. J. Mater. Chem. A 10 (2022) 16115-16126.

[3] Y. Chen, G. Meng, T. Yang, C. Chen, Z. Chang, F. Kong, H. Tian, X. Cui, X. Hou, J. Shi, Interfacial engineering of Co-Doped 1T-MoS_2 coupled with V_2C MXene for efficient electrocatalytic hydrogen evolution. Chem. Eng. J 450 (2022) 138157.

[4] G. Wang, G. Zhang, X. Ke, X. Chen, Y. Wang, G. Huang, J. Dong, S. Chu, M. Sui, Direct synthesis of stable 1T-MoS_2 doped with Ni single atoms for water splitting in alkaline media. Small 18 (2022) e2107238.

[5] Z. Zhao, Z. Li, Z. Zhang, X. Meng. Fe/p dual-doping $NiMoO_4$ With hollow structure for efficient hydrazine oxidation-assisted hydrogen generation in alkaline seawater. Appl. Catal. B: Environ: 347(2024)123805.

[6] H. Hu, Z. Zhang, Y. Zhang, T. Thomas, H. Du, K. Huang, J. Paul Attfield, M. Yang, An ultra-low Pt metal nitride electrocatalyst for sustainable seawater hydrogen production. Energy. Environ. Sci. 16 (2023) 4584-4592.

[7] H. Zhang, J. Diao, M. Ouyang, H. Yadegari, M. Mao, M. Wang, G. Henkelman, F. Xie, Heterostructured core-shell Ni-Co@Fe-Co nanoboxes of prussian blue analogues for efficient electrocatalytic hydrogen evolution from alkaline seawater. ACS Catal.13 (2023) 1349-1358.

[8] Y. Song, M. Sun, S. Zhang, X. Zhang, P. Yi, J. Liu, B. Huang, M. Huang, L. Zhang. Alleviating the work function of Vein-Like Co_xP by Cr doping for enhanced seawater electrolysis. Adv. Funct. Mater. 33 (2023) 2214081.

[9] J. Lu, S. Chen, Y. Zhuo, X. Mao, D. Liu, Zhen. Wang, Greatly boosting seawater hydrogen evolution by surface amorphization and morphology engineering on $MoO_2/Ni_3(PO_4)_2$. Adv. Funct. Mater. 33 (2023) 2308191.

[10] J. Gupta, D. Das, P. H, Borse, S.V. Bulusu. In-situ Pd doped MoS_2 nanosheets as HER electrocatalyst for enhanced electrocatalytic water splitting. Sustainable Energy Fuels 8 (2024) 1526-1539.

[11] K. Wu, K. Sun, S. Liu, W. Cheong, Z. Chen, C. Zhang, Y. Pan, Y. Pan, Y. Cheng, Z. Zhuang, X. Wei, Y. Wang, L. R. Zheng, Q. H. Zhang, D. S. Wang, Q. Peng, C. Chen, Y. D. Li, Atomically dispersed Ni-Ru-P interface sites for high-efficiency pH-universal electrocatalysis of hydrogen evolution. Nano Energy 80 (2021)105467.
